# Reinforcement of Thermoplastic Corn Starch with Crosslinked Starch/Chitosan Microparticles

**DOI:** 10.3390/polym10090985

**Published:** 2018-09-04

**Authors:** Diana Paiva, André M. Pereira, Ana L. Pires, Jorge Martins, Luísa H. Carvalho, Fernão D. Magalhães

**Affiliations:** 1 LEPABE, Faculdade de Engenharia da Universidade do Porto, Rua Dr. Roberto Frias, 4200-465 Porto, Portugal; diana.paiva@fe.up.pt (D.P.); jmmartins@estv.ipv.pt (J.M.); lhcarvalho@estv.ipv.pt (L.H.C.); 2 IFIMUP and IN—Institute of Nanoscience and Nanotechnology, Departamento de Física e Astronomia, Faculdade de Ciências, Universidade do Porto, 4169-007 Porto, Portugal; ampereira@fc.up.pt (A.M.P.); ana.pires@fc.up.pt (A.L.P.); 3 DEMad—Instituto Politécnico de Viseu, Campus Politécnico de Repeses 3504-510 Viseu, Portugal

**Keywords:** thermoplastic starch, corn starch, chitosan, crosslinked microparticles

## Abstract

Microparticles of corn starch and chitosan crosslinked with glutaraldehyde, produced by the solvent exchange technique, are studied as reinforcement fillers for thermoplastic corn starch plasticized with glycerol. The presence of 10% *w*/*w* chitosan in the microparticles is shown to be essential to guaranteeing effective crosslinking, as demonstrated by water solubility assays. Crosslinked chitosan forms an interpenetrating polymer network with starch chains, producing microparticles with a very low solubility. The thermal stability of the microparticles is in agreement with their polysaccharide composition. An XRD analysis showed that they have crystalline fraction of 32% with V_a_-type structure, and have no tendency to undergo retrogradation. The tensile strength, Young’s modulus, and toughness of thermoplastic starch increased by the incorporation of the crosslinked starch/chitosan microparticles by melt-mixing. Toughness increased 360% in relation to unfilled thermoplastic starch.

## 1. Introduction

Environmental concerns have increased public awareness over the use and disposal of common petroleum-based plastics, especially those used in manufacturing short-lifetime products, like disposable eating utensils, food packaging, bags, and so on. [1,2,3]. Bioplastics, sourced from natural materials, have been the growing focus of attention, as they are based on renewable raw materials and are biodegradable. Currently, the most widely used bioplastic is thermoplastic starch (TPS), either alone or blended with natural or synthetic polymers. TPS has some limitations, mainly related to high hydrophilicity, low processability, and a tendency to increase brittleness with time due to recrystallization (retrogradation) [4,5].

Native starch is composed of amylose, a linear polymer of d-glucose units linked by (1→4) bonds, and amylopectin, a highly branched polymer of d-glucose units linked by (1→4) bonds, branched with (1→6) linkages at intervals of approximately 20 units [6,7,8]. Starch granules have amorphous and crystalline fractions composed mostly of amylose and amylopectin, respectively [9,10].

The production of thermoplastic starch (TPS) requires the disruption of starch granules, in a process called gelatinization. Water or another solvent able to form hydrogen bonds with the starch chains is used, in conjunction with heat. As the amylose molecules dissolve and the amylopectin crystallites melt, a gelatinous paste is obtained [11,12,13]. After drying, the previously gelatinized starch is not processable, because of strong intermolecular hydrogen bonding in amylose and amylopectin chains. Plasticizers must therefore be used to allow for processing by extrusion or injection molding. Common starch plasticizers are water, glycerol, sorbitol, and urea [14,15,16,17]. As the plasticizer content increases in TPS, so does the elongation at break, but the stiffness and tensile resistance decrease [18]. Depending on the desired application, the amount of plasticizer must be adjusted in order to achieve the intended performance. The source of the starch will also be determinant for the mechanical properties of TPS, as it affects the amylose/amylopectin ratio and thus the crystallinity of the material [19]. Starches with high amylose content typically crystallize to a higher extent after processing, and thus have a higher tensile strength and lower elongation at break [20,21,22].

Lignocellulosic fibers are known to provide good mechanical reinforcement in TPS [23]. Examples include sisal, hemp and green coconut fibers [24,25], cotton cellulose nanofibers [26], and cellulose fibers from recycled paper [27]. Particulate fillers, like graphene [28], calcium carbonate nanoparticles [29], and clays [30,31] have also been reported to improve the mechanical properties of TPS.

In this work, a new approach for reinforcing thermoplastic corn starch is studied, which is based on the incorporation of starch/chitosan microparticles crosslinked with glutaraldehyde. The fact that the filler particles are of the same nature as the matrix should ensure a good compatibility between the two, and provide strong interfacial interactions. The filler microparticles are intended to be mechanically tough, thus taking advantage of the known affinity between starch and chitosan, and the ability of dialdehydes to chemically crosslink these blends [32].

## 2. Materials and Methods

### 2.1. Materials

The corn starch was purchased from Frutalcarmo (Alcoentre, Portugal), chitosan ([CS] degree of acetylation 90%) from Golden-Shell Pharmaceutical Co., Ltd. (Yuhuan County, Zhejiang, China), glutaraldehyde solution (25% in H_2_O) and propionic acid from Sigma-Aldrich (Lisbon, Portugal), glycerol from JMGS (Odivelas, Portugal), ethanol from PanReac AppliChem (Madrid, Spain), and hydrochloric acid from Fisher (Porto Salvo, Portugal). All of the materials were used as purchased, unless otherwise noted.

### 2.2. Preparation of Crosslinked Starch/Chitosan Microparticles

The starch/chitosan microparticles (SCM) were prepared using the solvent exchange technique, adapting procedures previously described in the literature for microparticles composed solely of starch [6,33]. The first step consists of the gelatinization of corn starch in order to disrupt the granules. 8 g (0.049 mol) of polysaccharide (either starch alone, or a mixture of 90% *w*/*w* starch and 10% *w*/*w* chitosan) was added to 25 mL of distilled water under mechanical agitation (Heidolph RZR2041, 300 rpm, Schwabach, Germany). To ensure complete dissolution, chitosan was added in the form of an acidic aqueous solution with 5% chitosan and 6% propionic acid. The aqueous mixture of the starch and chitosan was heated to 80 °C, the vessel’s external jacket was connected to a recirculating thermostatic bath for a period of 20 min, and was kept at that temperature for an additional 20 min, under mild agitation. The obtained gel was then cooled to 50 °C over a period of 1 h. Then, 100 mL of ethanol was added and the mechanical stirring increased to 700 rpm and was maintained for 15 min. A visibly agglomerated precipitate was formed. In order to promote deagglomeration, the dispersion was kept for 15 min under high shear, using an IKA T-18 Ultra-turrax (Staufen, Germany) at 10,000 rpm. The precipitate was then filtered using qualitative filter paper (5–13 µm, VWR international, Radnor, PA, USA, Grade 413) in a Buchner funnel under a vacuum, in order to remove the excess water. To promote crosslinking, the particles were re-suspended in 100 mL of 90% (*v*/*v*) ethanol and the dispersion was heated to 50 °C under mechanical stirring (500 rpm). Glutaraldehyde was added in different amounts, from 0 to 10 g per 100 g of polysaccharide, mixed with 0.5 mL of HCl 1 M, after which the dispersion was kept at 50 °C for 1 h. The resulting particles were filtered as described above and re-suspended in 100 mL of ethanol under mechanical stirring at 300 rpm for a period of 15 min, to remove any unreacted glutaraldehyde. The particles were finally filtered and dried to constant weight at 105 °C.

### 2.3. Preparation of Thermoplastic Starch

The thermoplastic materials were prepared by melt mixing, using 30% glycerol as a plasticizer, as described by Carvalho et al. [34]. The SCM content was varied from 0 to 50%. Dried native corn starch granules and glycerol were pre-mixed the day before use, to promote the absorption of glycerol by the granules. This pre-mixture and the intended amount of SCM were fed to a DSM Xplore 5 twin screw microcompounder (Sittard, The Netherlands), having a 5.5 mL conical barrel and recirculation channel. The mixing chamber temperature was 140 °C. The twin screws were operated at 200 rpm and the mixture time after loading was 5 min. The resulting mixture was then injected into a mold, using a DSM 10 cc micro injection molder (Sittard, The Netherlands), in order to produce dog-bone shaped specimens with a 2 mm thickness and 80 mm length. The injection pressure was 10 bar, the temperature in the injection nozzle was set to 150 °C, and the mold temperature to 60 °C.

### 2.4. Characterization

The water solubility and boiling water solubility of the SCM were evaluated in terms of the total soluble matter (TSM). Both tests were performed in an aqueous solution with two pH values (4 and 7). The water solubility was evaluated for a period of 24 h under constant magnetic stirring. The boiling water solubility was evaluated for a period of 1 h. Previously dried and weighted particles were used. After the conclusion of the tests, the liquid was filtered and the residue was dried to a constant weight. The TSM value, in a percentage, is given by Equation (1), where *m_final_* is the final mass after drying and *m_initial_* is the initial mass of the particles, as follows:

(1) $$\;\mathrm{TSM}\;(\%)\;=1-\frac{m_{final}}{m_{initial}}\times 100$$

An FTIR analysis of the native starch and microparticles was performed on a VERTEX 70 FTIR spectrometer (BRUKER, Billerica, MA, USA) in absorbance mode, with a high sensitivity DLaTGS detector at room temperature. The samples were measured in ATR mode, with a A225/Q PLATINUM ATR Diamond crystal (Billerica, MA, USA) with single reflection accessory. The spectra were recorded from 4000 to 500 cm^−1^ with a resolution of 4 cm^−1^.

X-ray diffraction (XRD) measurements were performed on native cornstarch granules and on SCM crosslinked with 7.5% glutaraldehyde, after one day and 30 days after production. The experiments were performed at the IFIMUP-IN facilities, in a Rigaku SmartLab diffractometer (Tokyo, Japan) that operates with 9 kW power (45 kV and 200 mA) and a Cu source with a wavelength *λ* = 1.540593 Å in Bragg-Brentano geometry. All of the samples were measured at room temperature over the range 2*θ* = 5–30° in rotation mode.

The mean diameter of crystallite was calculated with the Debye-Scherrer equation, as follows:

(2) $$\;D_{hkl\;}=\frac{K\lambda }{B_{hkl}cos\theta }$$

where *D_hkl_* is considered on the direction perpendicular to the lattice planes, *hkl* is the Miller indices of the planes being analysed, *λ* is the wavelength of the source Cu K*α* (*λ* = 1.5406 Å), *B_hkl_* is the full-width at half-maximum (FWHM) of the principal peak, and (002) *θ* is the Bragg angle [35].

The volumetric crystallinity fraction was determined accordingly to the following equation [36]:

(3) $$\;\mathrm{Crystallinity}(\%)\;=\frac{I_{T}-I_{A}}{I_{T}}\times 100$$

where *I_T_* is the total area under the intensity curve and *I_A_* is the area under the amorphous halo.

The FWHM/area determinations were performed using the free license Fityk software (version 0.9.8) [37].

The morphological characterization of SCM was performed using a scanning electron microscope (SEM, Hillsboro, OR, USA), FEI QUANTA 400 FEG ESEM/EDAX Pegasus X4M, property of CEMUP-Centro de Materiais da Universidade do Porto. The sample was placed on carbon tape and coated with a gold–palladium (Au-Pd) layer to ensure conductivity, and then analyzed at a voltage of 15 kV.

The thermogravimetry analysis (TGA) of SCM was performed in a STA 449 F3 Jupiter (Netzsch, Selb, Germany). The samples weights were about 10 mg. The runs were carried out from 30 to 550 °C, at a rate of 10 K·min^−1^, in aluminum pans under a nitrogen flow.

The tensile tests of the composite thermoplastic starch specimens were performed in a Tinius Olsen H50KT universal tensile testing machine equipped with a load cell of 10 kN at crosshead speed of 2 mm/min, according to the standard of ISO 527-1. The tension tests were conducted at ambient conditions (20 °C, 65% relative humidity) on dog-bone shaped samples (80 mm × 11 mm × 2 mm), according to ISO 527-2. The ultimate tensile strength, percentage elongation at break, and tensile modulus values were recorded using the software Tinius Olsen Horizon, according to ISO 527-1.

Water absorption of thermoplastic starch was evaluated as described by Prachayawarakorn et al. [38]. The specimens were stabilized at room temperature for over a month. After stabilization, the samples from five different specimens of each composition were dried for 12 h in a vacuum oven at 50 °C and 125 mbar. The samples were then placed in a desiccator to cool to room temperature for 1 h, and then placed in a closed container at 100% relative humidity (RH). The samples were weighed after 8 h, 24 h, 3 days, 7 days, and 14 days, and the water absorption was computed according to the following equation:

(4) $$\;\%\;abs=\left(\frac{m_{wet\;}-m_{dry}}{m_{dry}}\right)\times 100$$

## 3. Results and Discussion

### 3.1. Starch/Chitosan Microparticles

**Figure 1:**
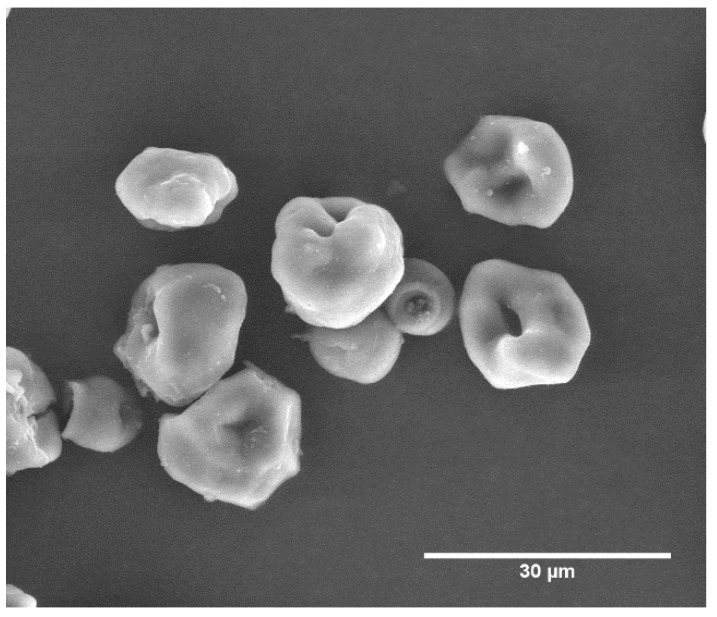
SEM image of starch/chitosan microparticles crosslinked with 7.5% of glutaraldehyde.

Figure 1 shows a representative SEM image of the crosslinked starch/chitosan microparticles (SCM). These microparticles have roughly spherical shapes with diameters between about 10 and 20 μm, independent on the amount of glutaraldehyde crosslinker used.

**Figure 2:**
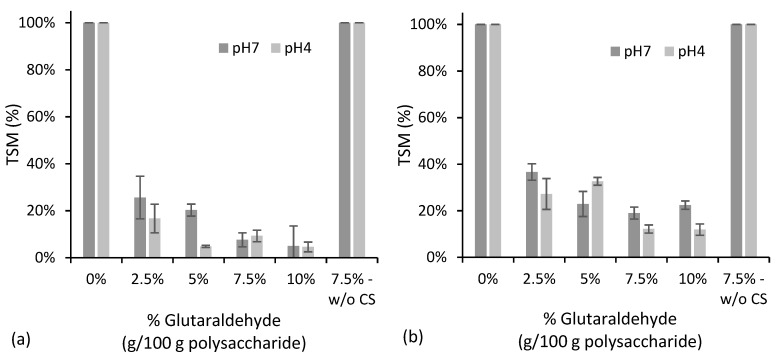
Water solubility at room temperature (**a**) and at boiling conditions (**b**), expressed in terms of total soluble matter (TSM), for starch/chitosan microparticles with different glutaraldehyde contents. All of the microparticles contain chitosan except the one labeled “w/o CS”.

The use of dialdehydes, such as glutaraldehyde or glyoxal, as crosslinking agents for polysaccharides is commonly mentioned in the literature [39,40,41]. A straightforward form of evaluating the effectiveness of the crosslinking reaction is by determining the product solubility. As new intermolecular covalent bonds have been formed, the water solubility is expected to decrease significantly. SCM with different amounts of glutaraldehyde were prepared, and the particles’ solubility was measured using two different assays, after 24 h immersion at room temperature, and after 1 h immersion in boiling water. As chitosan is insoluble at a neutral pH and completely soluble under acidic conditions (pH < 5), because of the hydrophilic character of the protonated amine groups, the particles’ solubility was evaluated for two pH values (7 and 4). Figure 2 presents the results obtained in terms of the total soluble matter (TSM).

The SCMs prepared without the glutaraldehyde addition are completely soluble at both pH values. The physical interaction between the starch and chitosan did not prevent solubility, even at a neutral pH. However, the addition of crosslinker greatly decreased the solubility. For both room temperature and boiling water immersion tests, increasing the glutaraldehyde concentration up to 7.5% tends to increase the water resistance. A further increase to 10% does not improve the results. Therefore, 7.5% seems to be an appropriate value for attaining effective crosslinking. TSM is higher when SCM is in contact with boiling water, as expected for such extreme conditions. Somewhat unexpectedly, however, TSM tends to be higher for pH 7 than for pH 4. The opposite could have been predicted, considering that chitosan is soluble only under an acidic pH. However, reaction of aldehydes with amines is known to be catalyzed by acids. Therefore, the lower TSM observed at pH 4 is probably a consequence of further crosslinking taking place between residual unreacted glutaraldehyde and amino groups in chitosan. When chitosan is not used, the microparticles become completely soluble, despite of the presence of glutaraldehyde. This suggests that only chitosan’s amino groups, and not starch’s hydroxyl groups, intervene in the crosslinking process with the aldehyde.

**Figure 3:**
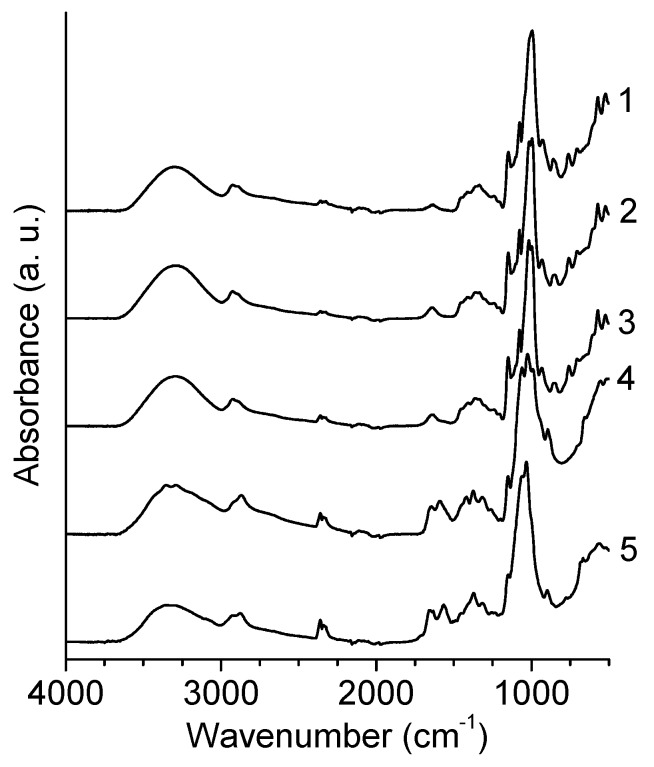
FTIR spectra of: (1) native starch, (2) starch + 7.5% glutaraldehyde, (3) starch + 10% chitosan + 7.5% glutaraldehyde, (4) chitosan, and (5) chitosan + 7.5% glutaraldehyde.

A FTIR analysis was performed on native starch and on starch microparticles containing 7.5% glutaraldehyde, both with and without the chitosan addition. The results are show in Figure 3. The microparticles without chitosan did not present significant changes on the spectra when compared to the native starch, which may be an indication that the reaction between the starch and glutaraldehyde is unlikely. Because of its low concentration, the addition of chitosan to the microparticles was not detectable by FTIR, as the typical bands of NH_2_ group (1650–1580 cm^−1^) are not present in the corresponding spectrum. As a consequence, the bands that would result from the reaction of chitosan with glutaraldehyde are also not detectable. Nonetheless, in order to demonstrate the feasibility of this chemical reaction under the conditions used for production of the microparticles, the same synthesis procedure was followed using chitosan alone, with and without the glutaraldehyde addition. The resulting spectra are also shown in Figure 3. When glutaraldehyde is added to chitosan, a new band appears at 1658 cm^−1^, confirming the formation of the N=C linkage, originated by the reaction between an amine group from chitosan and an aldehyde group from glutaraldehyde. In addition, a small band around 1720 cm^−1^ is visible, which may be attributed to the unreacted aldehyde groups from glutaraldehyde. It must be noted that glutaraldehyde was already known to be an efficient crosslinker for chitosan [42,43]. It can therefore be suggested that the crosslinked SCM particles consist of an interpenetrating polymer network of starch and crosslinked chitosan chains.

**Figure 4:**
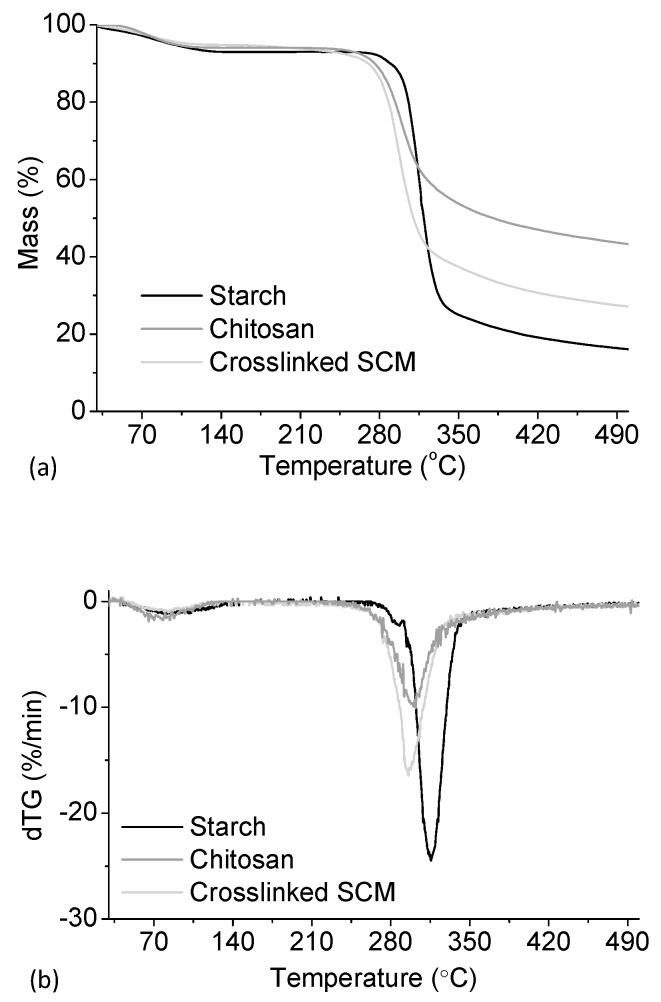
Mass loss (**a**) and first derivative (**b**) for native corn starch, chitosan, and starch/chitosan microparticles (SCM) crosslinked with 7.5% of glutaraldehyde. The thermogravimetric runs were performed under nitrogen atmosphere.

Thermogravimetric curves were obtained for the native corn starch, chitosan, and SCM crosslinked with 7.5% glutaraldehyde. These are shown in Figure 4.

All of the materials show an initial mass loss consistent with the desorption of water molecules, corresponding to roughly 6% of the original mass. The onset of thermal degradation for corn starch is 311 °C, which is consistent with the literature [44,45]. On the other hand, for chitosan, the onset occurs earlier, at 280 °C, and the mass fraction remaining at 500 °C is much higher than for starch (41% compared to 16%). The onset of degradation for crosslinked SCM occurs at the same temperature as for chitosan, and the residual mass fraction is 27%. Considering the starch/chitosan ratio (9:1) present in the microparticles, and the residual mass fraction measured for each single compound, a residual mass of 19% would be expected for the microparticles. The higher value obtained is a consequence of crosslinking with glutaraldehyde, improving thermal stability of the material.

**Figure 5:**
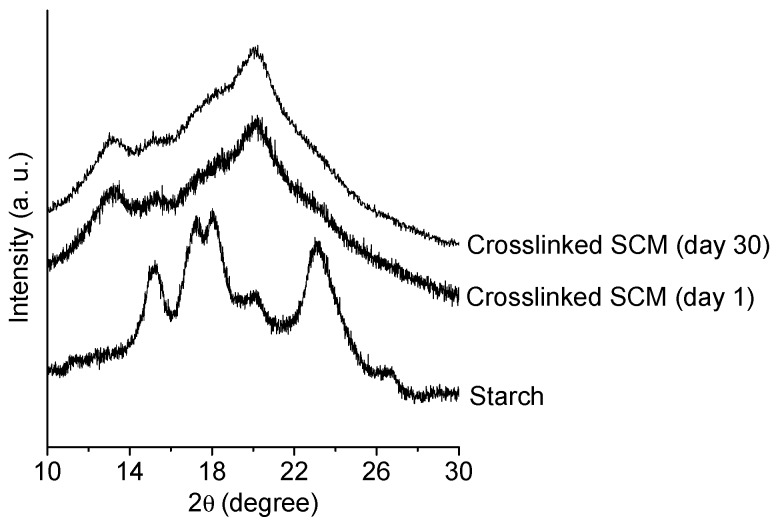
X-ray diffraction patterns for native corn starch and starch/chitosan microparticles with 7.5% of glutaraldehyde (crosslinked SCM), at day 1 and day 30 after production.

X-ray diffraction measurements were performed on native cornstarch granules and on SCM crosslinked with 7.5% glutaraldehyde. For the SCM, the XRD spectra were obtained one day and 30 days after production, in order to evaluate the possible changes in the crystallinity over time. A 30-day period is considered sufficient for retrogradation to occur in starch molecules after gelatinization [46,47,48]. Figure 5 presents the XRD diffractograms in the range 2*θ* = 10–30°.

Native corn starch exhibits diffraction peaks at 2*θ* = 15.1°, 17.6°, and 23.0°, consistent with a crystalline structure with A-type polymorphism that is usually found in cereal starches [49,50]. The A-type crystallites are, normally, denser and less hydrated because of the double-helical arrangement of amylopectin chains. One day after production, the starch/chitosan particles show peaks at 2*θ* = 13.2° and 20.2°, resembling the V_a_-type microstructure [18,51]. This polymorphism usually appears after gelatinization, and, with time, may undergo a transformation into V_h_-type crystals as a result of exposure to humidity. This process is called retrogradation and is associated with an increase in the brittleness of the starch material [52,53]. The XRD pattern obtained for the same particles after 30 days does not exhibit the peak characteristic of the V_h_-type structure (2*θ* = 18.3°), indicating that the V_a_-type crystalline structure is maintained. Therefore, one may conclude that crosslinking with glutaraldehyde after gelatinization/precipitation induces the stability of the crystalline structure, hindering the retrogradation of starch chains. The mean diameter of the crystals was determined by the Debye-Scherrer equation (Equation (2)), and the fraction of crystallinity by peak area integration (Equation (3)). In native starch, the crystals presented diameters around 6.7 nm and a 55% crystallinity. The crosslinked particles presented a smaller crystal mean diameter and lower crystallinity of 4.1 nm and 32%, respectively, on day 1. On day 30, the crystal size and fraction of crystallinity were similar, as expected in the absence of retrogradation.

### 3.2. Reinforced Thermoplastic Starch

**Figure 6:**
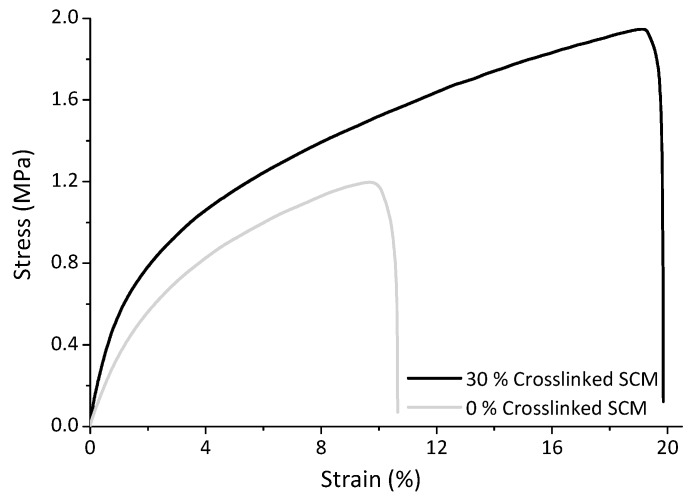
Representative stress-strain curve obtained for thermoplastic starch containing 30% SCM crosslinked with 7.5% glutaraldehyde and without SCM.

Thermoplastic starch samples containing different amounts of SCM crosslinked with 7.5% glutaraldehyde were produced by melt mixing followed by injection molding. Their mechanical performance was evaluated in stress-strain tests. Figure 6 shows representative examples of the curves obtained.

The shape of the stress-strain curves indicates that linear elastic behavior is present only at low strains, and is followed by extensive ductile deformation, without a defined stress yield point. This behavior is typical of thermoplastic starches [19]. Strain hardening occurs during the plastic deformation regime, as a consequence of the reorientation of molecular chains and/or crystalline regions in the direction of the applied stress. This contributes to increase the toughness of the material under tensile strain [54].

**Figure 7:**
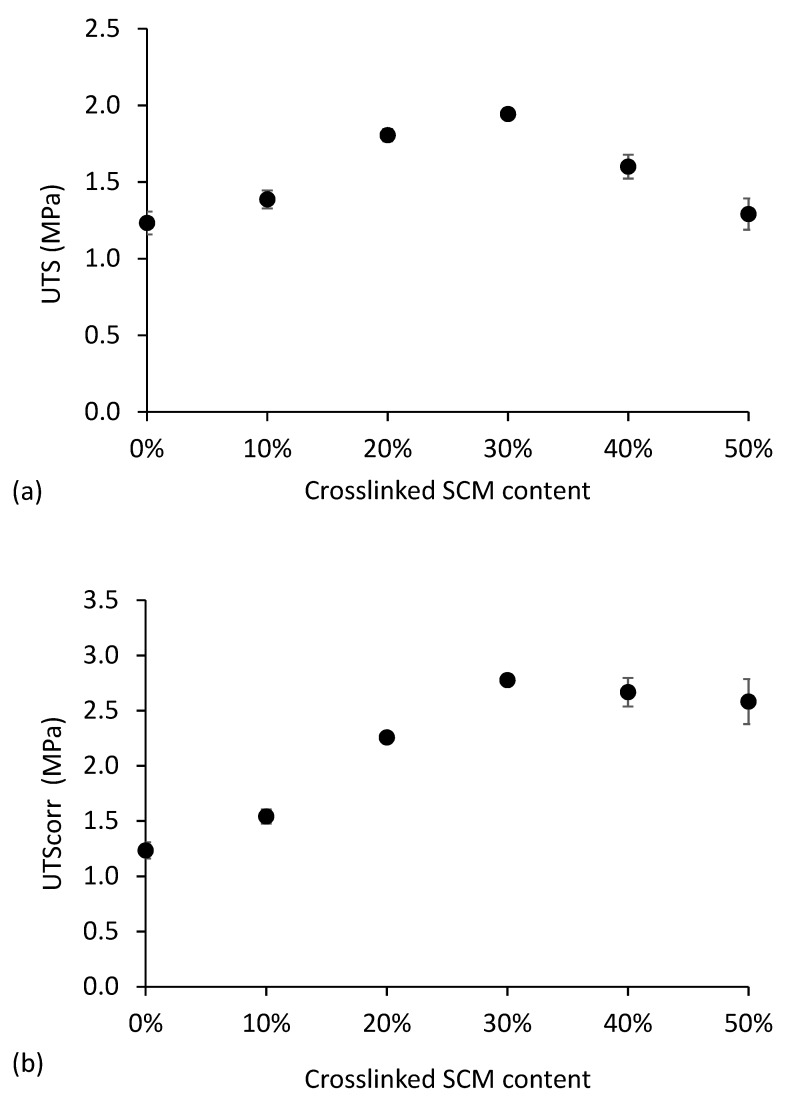
Ultimate tensile strength (UTS) (**a**) and corrected ultimate tensile strength (UTS_corr_) (**b**) as a function of crosslinked SCM content in thermoplastic starch.

The ultimate tensile strength (UTS) obtained for the thermoplastic starch samples is plotted in Figure 7a. Figure 7b represents the corrected ultimate tensile strength (UTS_corr_), that is, the UTS divided by the fraction of thermoplastic starch present in the sample. If the SCM acted as an inert filler, having no effect on mechanical reinforcement, UTS would decrease with the increasing SCM content, but the UTS_corr_ should remain constant.

Figure 7a shows that the UTS reaches a value 58% higher than the neat TPS for the 30% SCM content, and decreases afterwards. This reinforcement is a consequence of an efficient stress transfer through a strong interfacial bond between the thermoplastic matrix and the microparticles. As expected, concomitantly with the UTS decrease above the 30% SCM content, the UTS_corr_ stabilizes, showing that no additional reinforcement is obtained by incrementing the amount of SCM. This is probably due to microparticle agglomeration within the thermoplastic starch matrix, which does not contribute to an increase in the interfacial stress transfer.

**Figure 8:**
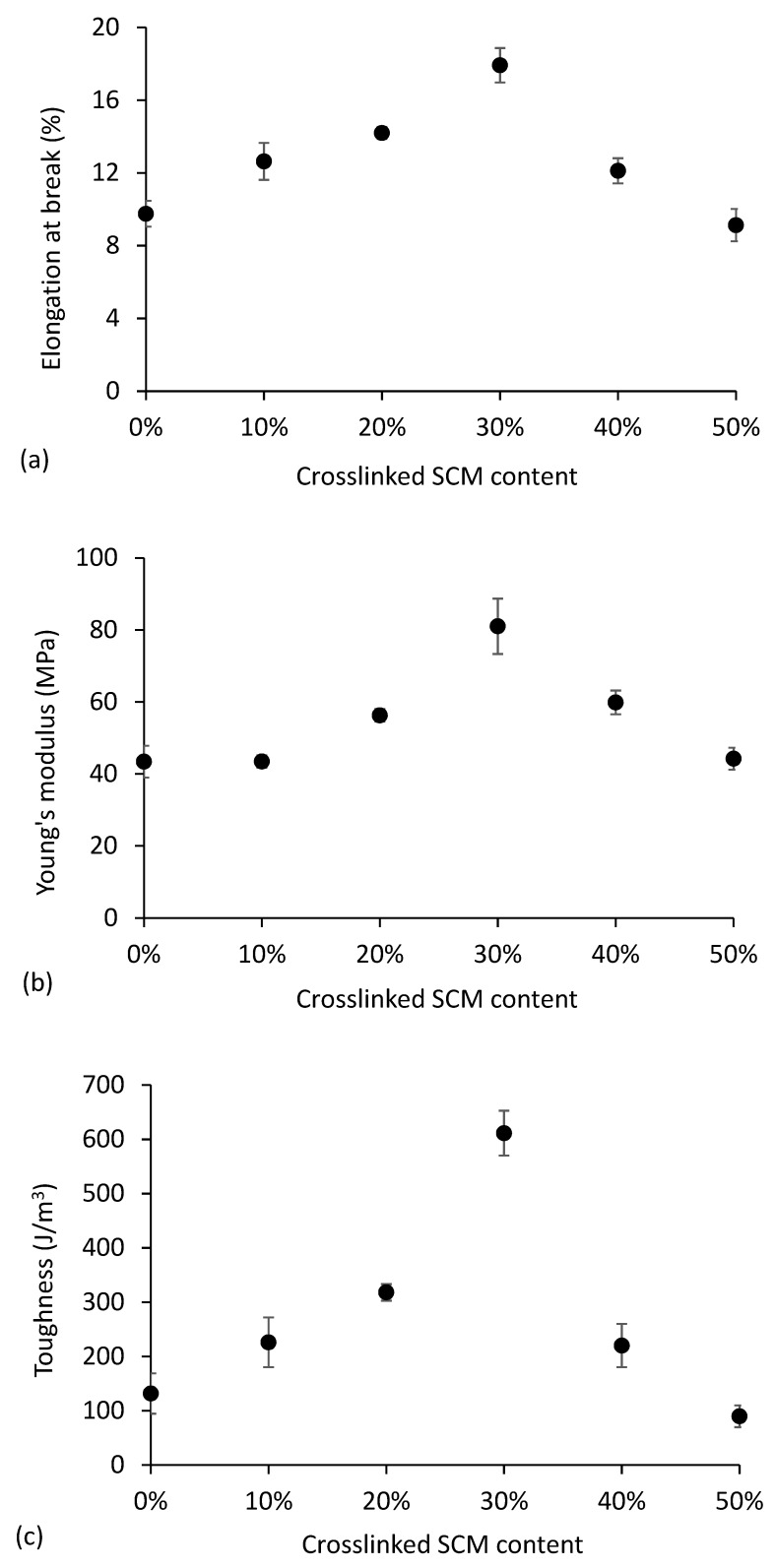
Elongation at break (**a**), Young’s modulus (**b**), and toughness (**c**) as a function of crosslinked SCM content in thermoplastic starch.

Figure 8 presents the elongation at break, Young’s modulus, and toughness for all of the materials. All of the properties exhibit a maximum for the 30% SCM content. The initial increase in elongation at break with filler content, seen in Figure 8a, is not the most common behavior. Reinforcement with a filler usually translates into a continuous decrease in elongation at break, concomitantly with an increase in rigidity (Young’s modulus), as the chain mobility is restrained by matrix-filler interactions [24]. The observed 84% increase may be due to the microparticles being able to undergo elastic deformation while maintaining strong physical bonding with the thermoplastic matrix. The work of Kvien and co-workers with potato starch plasticized with sorbitol and filled with cellulose nanowhiskers also evidenced an increase in elongation at break with the filler content. The authors proposed that the interaction of the nanofibers with the plasticizer could maximize the plasticizer effect as well as reinforce the Young’s modulus of the material [55]. Teixeira and co-workers reported a 66% increase in elongation at break with the incorporation of 5% cotton cellulose nanofibers in thermoplastic corn starch [26].

The combined increase in rigidity, strength, and elongation at break for the SCM contents up to 30% leads to a significant improvement in toughness, computed as the area under the stress-strain curves. Toughness is about 360% higher for the 30% SCM content than for the neat thermoplastic starch, which translates into a much higher capacity to absorb energy without fracture. Above this filler content, all of the properties decrease, due to an inefficient dispersion of the microparticles, as mentioned before. Agglomerates behave as defects with lower cohesion than the rest of the material.

**Figure 9:**
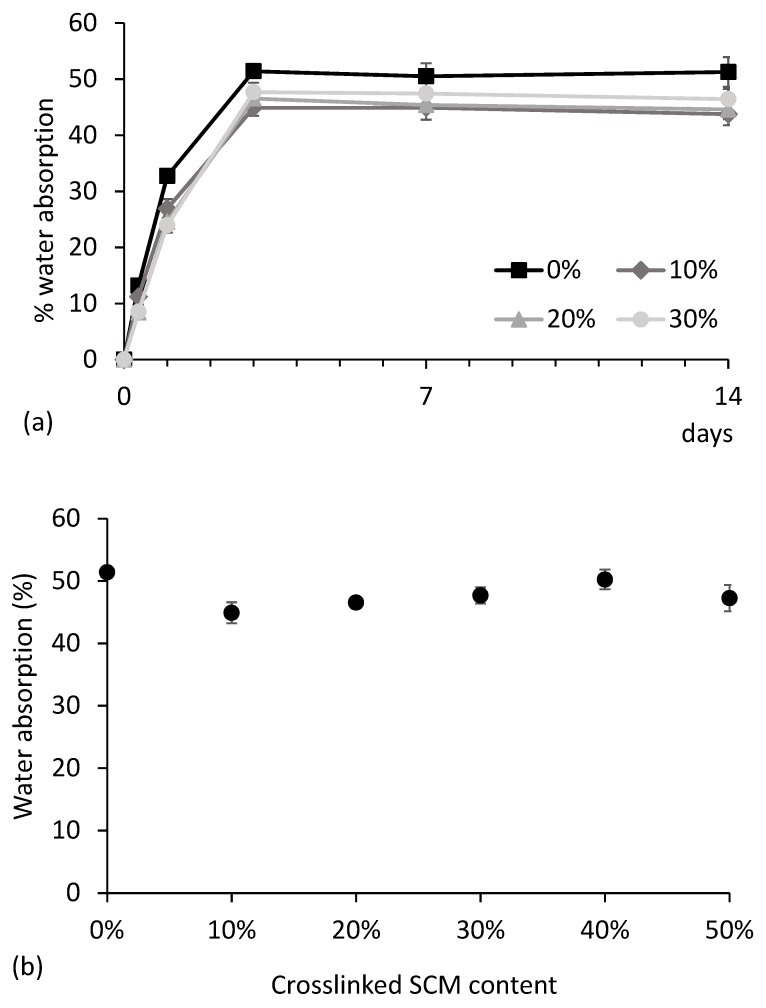
Examples of water uptake at 100% relative humidity for some crosslinked SCM contents in thermoplastic starch (**a**), and equilibrium water absorption after 3 days as a function of crosslinked SCM content (**b**).

The water absorption was measured along the time under 100% relative humidity for all of the materials (Figure 9). In all cases, the maximum absorption is attained after three days. The kinetics of the water uptake is similar to that previously reported for thermoplastic corn starch reinforced with wood fiber [56]. The equilibrium absorption values, shown in Figure 9b, show that there is not a clear relation with the SCM content. The variations observed are probably due to an inaccuracy of the measurement method. The microparticles, despite being insoluble, are able to absorb water and therefore do not seem to affect the hydrophilicity of the material. Other researchers have reported some reduction in the water uptake for some types of fillers. As an example, glycerol-plasticized potato starch reinforced with 30% cellulose microfibrils had water uptake 14% lower than unfilled starch [57].

## 4. Conclusions

Microparticles composed of corn starch and chitosan were produced by solvent exchange and crosslinked with glutaraldehyde. The effectiveness of crosslinking was evaluated by measuring the fraction of soluble matter. The results showed that the microparticles are completely soluble when composed of only starch. When 10% *w*/*w* chitosan is used, the soluble fraction becomes relatively low, tending to decrease as the crosslinker content is increased. For 7.5 g glutaraldehyde per 100 g of polysaccharide, the total soluble matter is lower than 10% after one day of immersion in water at room temperature, and lower than 20% after one hour in boiling water, even under acidic conditions that facilitate the chitosan dissolution. The presence of chitosan’s amino groups is therefore essential for a reaction with glutaraldehyde, forming an interpenetrating polymer network with starch chains. Thermogravimetry indicated that the microparticles’ thermal degradation behavior is coherent with their polysaccharide composition. An XRD analysis showed that the microparticles have 32% crystalline fraction with V_a_-type structure, and do not exhibit a tendency to undergo retrogradation.

The crosslinked starch/chitosan microparticles were melt-mixed with corn starch plasticized with glycerol and were able to reinforce its mechanical properties in terms of tensile strength, rigidity, and toughness. Contrary to what is usual, elongation at break also increases with the filler content, probably due to the microparticles being able to deform while providing an interfacial stress transfer. An optimum weight fraction of 30% was identified, leading to 58%, 87%, and 84% increases in ultimate tensile strength, Young’s modulus, and elongation at break, respectively. The materials toughness increased 360% in relation to unfilled thermoplastic starch.

## References

[1] Shogren R.L., Swanson C.L., Thompson A.R. (1992). Extrudates of cornstarch with urea and glycols: Structure/mechanical property relations. Starch-Stärke.

[2] Stevens E.S., Klamczynski A., Glenn G.M. (2010). Starch-lignin foams. Express Polym. Lett..

[3] Patel S.V., Venditti R.A., Pawlak J.J. (2009). Dimensional changes of starch microcellular foam during the exchange of water with ethanol and subsequent drying. BioResources.

[4] Jiankang W., Ruilin M., Liang H. (2017). Recent research and patents on preparation and application of starch-based plastics. Recent Patents Mech. Eng..

[5] Carvalho A.J.F. (2016). Chapter 6: Chemical Modification of Thermoplastic Starch. RSC Green Chemistry.

[6] Patel S., Venditti R.A., Pawlak J.J., Ayoub A., Rizvi S.S.H. (2009). Development of cross-linked starch microcellular foam by solvent exchange and reactive supercritical fluid extrusion. J. Appl. Polym. Sci..

[7] El-Tahlawy K., Venditti R., Pawlak J. (2008). Effect of alkyl ketene dimer reacted starch on the properties of starch microcellular foam using a solvent exchange technique. Carbohydr. Polym..

[8] Bastioli C., Magistrali P., Garcia S.G., Kabasci S. (2013). Starch. Bio-Based Plastics: Materials and Applications.

[9] Soykeabkaew N., Thanomsilp C., Suwantong O. (2015). A review: Starch-based composite foams. Compos. Part A Appl. Sci. Manuf..

[10] Blazek J., Gilbert E.P. (2011). Application of small-angle X-ray and neutron scattering techniques to the characterisation of starch structure: A review. Carbohydr. Polym..

[11] Carvalho A.J.F., Gandini A. (2008). Starch: Major Sources, Properties and Applications as Thermoplastic Materials a2—Belgacem, Mohamed Naceur. Monomers, Polymers and Composites from Renewable Resources.

[12] Wang S., Li C., Copeland L., Niu Q., Wang S. (2015). Starch retrogradation: A comprehensive review. Compr. Rev. Food Sci. Food Saf..

[13] Mihai M., Huneault M.A., Favis B.D., Li H. (2007). Extrusion foaming of semi-crystalline PLA and PLA/thermoplastic starch blends. Macromol. Biosci..

[14] Mihai M., Huneault M.A., Favis B.D. (2007). Foaming of polystyrene/thermoplastic starch blends. J. Cell. Plast..

[15] Chen M., Chen B., Evans J.R.G. (2005). Novel thermoplastic starch-clay nanocomposite foams. Nanotechnology.

[16] Karger-Kocsis J., Kmetty Á., Lendvai L., Drakopoulos S., Bárány T. (2015). Water-assisted production of thermoplastic nanocomposites: A review. Materials.

[17] Bahram K., Muhammad B.K.N., Ghufrana S., Zaib J. (2017). Thermoplastic starch: A possible biodegradable food packaging material—A review. J. Food Process Eng..

[18] Esmaeili M., Pircheraghi G., Bagheri R. (2017). Optimizing mechanical and physical properties of thermoplastic starch via tuning the molecular microstructure through co-plasticization by sorbitol and glycerol. Polym. Int..

[19] Graaf R.A., Karman A.P., Janssen L.P.B.M. (2003). Material properties and glass transition temperatures of different thermoplastic starches after extrusion processing. Starch-Stärke.

[20] Mali S., Grossmann M.V.E., García M.A., Martino M.N., Zaritzky N.E. (2006). Effects of controlled storage on thermal, mechanical and barrier properties of plasticized films from different starch sources. J. Food Eng..

[21] Xie F., Luckman P., Milne J., McDonald L., Young C., Tu C.Y., Pasquale T.D., Faveere R., Halley P.J. (2014). Thermoplastic starch: Current development and future trends. J. Renew. Mater..

[22] Salam A., Pawlak J.J., Venditti R.A., El-tahlawy K. (2010). Synthesis and characterization of starch citrate−chitosan foam with superior water and saline absorbance properties. Biomacromolecules.

[23] Gutiérrez T.J., Alvarez V.A. (2017). Cellulosic materials as natural fillers in starch-containing matrix-based films: A review. Polym. Bull..

[24] Gironès J., López J.P., Mutjé P., Carvalho A.J.F., Curvelo A.A.S., Vilaseca F. (2012). Natural fiber-reinforced thermoplastic starch composites obtained by melt processing. Compos. Sci. Technol..

[25] Lomelí-Ramírez M.G., Kestur S.G., Manríquez-González R., Iwakiri S., de Muniz G.B., Flores-Sahagun T.S. (2014). Bio-composites of cassava starch-green coconut fiber: Part ii—Structure and properties. Carbohydr. Polym..

[26] Teixeira E., Lotti C., Corrêa A.C., Teodoro K.B.R., Marconcini J.M., Mattoso L.H.C. (2011). Thermoplastic corn starch reinforced with cotton cellulose nanofibers. J. Appl. Polym. Sci..

[27] Wattanakornsiri A., Pachana K., Kaewpirom S., Traina M., Migliaresi C. (2012). Preparation and properties of green composites based on tapioca starch and differently recycled paper cellulose fibers. J. Polym. Environ..

[28] Jose J., Al-Harthi M.A., AlMa’adeed M.A.A., Dakua J.B., De S.K. (2015). Effect of graphene loading on thermomechanical properties of poly(vinyl alcohol)/starch blend. J. Appl. Polym. Sci..

[29] Kellermayer M.S., Sun Q., Xi T., Li Y., Xiong L. (2014). Characterization of corn starch films reinforced with caco3 nanoparticles. PLoS ONE.

[30] Chen B., Evans J.R.G. (2005). Thermoplastic starch–clay nanocomposites and their characteristics. Carbohydr. Polym..

[31] López O.V., Castillo L.A., García M.A., Villar M.A., Barbosa S.E. (2015). Food packaging bags based on thermoplastic corn starch reinforced with talc nanoparticles. Food Hydrocoll..

[32] Kumari K., Rani U. (2011). Controlled release of metformin hydrochloride through crosslinked blends of chitosan-starch. Adv. Appl. Sci. Res..

[33] El-Tahlawy K., Venditti R.A., Pawlak J.J. (2007). Aspects of the preparation of starch microcellular foam particles crosslinked with glutaraldehyde using a solvent exchange technique. Carbohydr. Polym..

[34] Carvalho A.J.F., Curvelo A.A.S., Agnelli J.A.M. (2001). A first insight on composites of thermoplastic starch and kaolin. Carbohydr. Polym..

[35] Holzwarth U., Gibson N. (2011). The scherrer equation versus the ‘debye-scherrer equation’. Nat. Nanotechnol..

[36] Jollet V., Chambon F., Rataboul F., Cabiac A., Pinel C., Guillon E., Essayem N. (2009). Non-catalyzed and pt/γ-Al_2_O_3_-catalyzed hydrothermal cellulose dissolution–conversion: Influence of the reaction parameters and analysis of the unreacted cellulose. Green Chem..

[37] Wojdyr M. (2010). Fityk: A general-purpose peak fitting program. J. Appl. Crystallogr..

[38] Prachayawarakorn J., Hanchana A. (2017). Effect of neem wood sawdust content on properties of biodegradable thermoplastic acetylated cassava starch/neem wood sawdust composites. Starch-Stärke.

[39] Mikkonen K.S., Heikkilä M.I., Willför S.M., Tenkanen M. (2012). Films from glyoxal-crosslinked spruce galactoglucomannans plasticized with sorbitol. Int. J. Polym. Sci..

[40] Patel A.K., Michaud P., de Baynast H., Grédiac M., Mathias J.-D. (2013). Preparation of chitosan-based adhesives and assessment of their mechanical properties. J. Appl. Polym. Sci..

[41] Yang Q., Dou F., Liang B., Shen Q. (2005). Investigations of the effects of glyoxal cross-linking on the structure and properties of chitosan fiber. Carbohydr. Polym..

[42] Monteiro O.A.C., Airoldi C. (1999). Some studies of crosslinking chitosan–glutaraldehyde interaction in a homogeneous system. Int. J. Biol. Macromol..

[43] Beppu M.M., Vieira R.S., Aimoli C.G., Santana C.C. (2007). Crosslinking of chitosan membranes using glutaraldehyde: Effect on ion permeability and water absorption. J. Membr. Sci..

[44] Liu X., Wang Y., Yu L., Tong Z., Chen L., Liu H., Li X. (2013). Thermal degradation and stability of starch under different processing conditions. Starch-Stärke.

[45] Liu X., Yu L., Liu H., Chen L., Li L. (2009). Thermal decomposition of corn starch with different amylose/amylopectin ratios in open and sealed systems. Cereal Chem..

[46] Ma X.F., Yu J.G., Wan J.J. (2006). Urea and ethanolamine as a mixed plasticizer for thermoplastic starch. Carbohydr. Polym..

[47] Zobel H.F., BeMiller J., Whistler R. (2009). Chapter IX—Gelatinization of Starch and Mechanical Properties of Starch Pastes. Starch: Chemistry and Technology.

[48] Arık Kibar E.A., Gönenç İ., Us F. (2011). Modeling of retrogradation of waxy and normal corn starches. Int. J. Food Prop..

[49] Cheetham N.W.H., Tao L.P. (1998). Variation in crystalline type with amylose content in maize starch granules: An X-ray powder diffraction study. Carbohydr. Polym..

[50] Zhang C.-W., Li F.-Y., Li J.-F., Wang L.-M., Xie Q., Xu J., Chen S. (2017). A new biodegradable composite with open cell by combining modified starch and plant fibers. Mater. Des..

[51] Van Soest J.J.G., Hulleman S.H.D., de Wit D., Vliegenthart J.F.G. (1996). Crystallinity in starch bioplastics. Ind. Crop Prod..

[52] Shanks R., Kong I., El-Sonbati P.A. (2012). Thermoplastic starch. Thermoplastic Elastomers.

[53] Nafchi A.M., Moradpour M., Saeidi M., Alias A.K. (2013). Thermoplastic starches: Properties, challenges, and prospects. Starch-Staerke.

[54] Roylance D. (1996). Mechanics of Materials.

[55] Kvien I., Sugiyama J., Votrubec M., Oksman K. (2007). Characterization of starch based nanocomposites. J. Mater. Sci..

[56] Carvalho A.J.F., Curvelo A.A.S., Agnelli J.A.M. (2002). Wood pulp reinforced thermoplastic starch composites. Int. J. Polym. Mater. Polym. Biomater..

[57] Dufresne A., Vignon M.R. (1998). Improvement of starch film performances using cellulose microfibrils. Macromolecules.

